# MIEBL: Measurement of Individualized, Evidence-Based Learning Criteria Designed for Discrete Trial Training

**DOI:** 10.1007/s40617-025-01058-9

**Published:** 2025-04-23

**Authors:** Mark Louie F. Ramos

**Affiliations:** Health Policy and Administration Department, College of Health and Human Development, The Pennsylvania State University, State College, PA USA

**Keywords:** Performance criterion, Discrete trial training, Bayesian inference, Probability, Tutorial

## Abstract

Informing the selection of a performance criterion for discrete trial training has been the subject of a growing body of empirical research, but an explicit framework has not yet been established. This paper proposes a tool for selecting a performance criterion that uses individualized assessment characteristics and mastery level goals and is grounded on sound probability theory. This tool is demonstrated to provide results that are consistent with existing research outcomes, and its use is advocated to better inform practitioners and researchers on the implications of their performance assessment choices. A tutorial with ready-to-use software is provided. Practitioners can use the tool to evaluate their assessment strategies and outcome expectations. Practitioners can use the tool to help make judgments about whether to continue with a teaching strategy or switch to another one. Researchers can use the tool to account for bias between observed performance and actual mastery level at the end of instruction, which is a confounder to observed performance during maintenance or generalization. Researchers and practitioners can use the tool to make better-informed decisions.

**Supplementary Information:**

The online version contains supplementary material available at 10.1007/s40617-025-01058-9.

## Introduction

Discrete trial training (DTT) is a structured teaching method used as a strategy in applied behavior analysis (ABA) therapy to help individuals with autism spectrum disorder (ASD) or other developmental disorders acquire practical skills (Smith, 2001). DTT breaks down the skill into its basic components and teaches each component intensively until the student can learn it enough to move on to the next component. Making this decision is an evidence-based task that typically involves examination of data on the student’s activities across multiple sessions and determining sufficiency based on the proportion of times that the student performed the task correctly out of all opportunities provided for the student to do so. If this proportion meets or exceeds some pre-specified threshold, then the desired mastery level is considered achieved. This threshold is referred to as the performance criterion, also called the mastery criterion, which has been the subject of research interest, especially more recently, with multiple experiments examining how changing the performance criterion impacts maintenance outcomes, which is how well the student retains the skills learned after instruction has ended (Wong et al., 2022a, 2022b). Despite this focus and many significant insights from these studies, a precise framework for DTT practice when it comes to decision-making about performance criterion remains elusive. Various studies have acknowledged the importance of such a framework, and the most recent information suggests there to be considerable variation both in what performance criterion is selected for practice and, more importantly, the rationale used to select the criterion. This paper proposes a flexible model for selecting performance criterion that is grounded on probability theory tailored to the individualized context of DTT.

### Development of Performance Criterion in DTT

The concept of performance criterion was present from the inception of DTT as a strategy for ABA (Baer et al., 1968; Lovaas, 1977), and at its core is the simple, intuitive idea that in order to infer whether a learner has learned content, one must observe how consistently the learner is able to answer questions on the content correctly. However, exactly how consistent this observation should be has been studied and widely debated for a long time. There is a wealth of literature on small sample experiments that infer how students’ ability to achieve a performance criterion during DTT sessions translates to their ability to demonstrate actual mastery over the content during follow-up/maintenance periods (Longino et al., 2022; Pitts & Hoerger, 2021; Wong et al., 2022a, 2022b). A systematic review conducted on 136 studies across three years found that performance criteria of 80% or higher tended to report corresponding maintenance and generalization outcomes, with studies that used higher criteria tending to report higher follow-up outcomes (Wong et al., 2022a, 2022b). Another study looked into the frequency of reaching the performance criterion and found similar maintenance levels when students needed to pass just one evaluation session at a 90% performance criterion using 15 trials and when they needed to pass three sessions (Schneider et al., 2022). One of the most recent handbooks for ABA practice discussed that different facets of assessment used to determine learning, including choosing the performance criterion, vary considerably across practitioners and researchers (Richling et al., 2023). Surveys conducted on practitioners found that some applied the criterion for trials in a single session, while others applied them to trials across multiple sessions (Richling et al., 2019). Richling et al. (2023) recommended that, at present, most existing literature agrees when it comes to performance criterion selection that it must be high, and they further recommend that it must be heavily nuanced to the skill in question, with some less critical skills being suitable for an 80% criterion while more critical ones that have greater impact when failed require much higher criterion.

### Measurement of Individualized, Evidence-Based Learning (MIEBL)

MIEBL provides an explicit framework for selecting performance criterion by applying probability theory. However, this application is nuanced to the context of DTT and, as such, differs from how probability and statistics are typically applied in the field of education. The usual application of probability and statistics in education is perhaps best exemplified by standardized testing, which seeks to identify and replicate optimal teaching methods based on comparing average performance across different schools and among different students (Gershon, 2015). In contrast to this, DTT practice is highly customized to individual students. As such, there is valid skepticism in using measures of average performance across the population of interest as a basis for performance criterion selection for a specific individual. Instead, MIEBL is focused on the individual student and offers a protocol for selecting performance criterion based only on factors concerning the individual. As will be explained, this does run into some difficulty in applying probabilistic concepts, particularly in terms of dealing with very small sample sizes, but MIEBL is designed to operate within those limitations.

We begin by defining $\Omega$ as a DTT skill component space. Its elements are items from a specific skill component that is desired for a student to learn. For example, ${\Omega }_{1}$ can be the DTT skill component space for recognizing and uttering the sight word “house” under some defined conditions (e.g., level of distraction, presence of prompting, motivating operations). The elements of ${\Omega }_{1}$ can be any trial used by the practitioner in determining whether the student can accomplish this component. Thus, the process of preparing a session where the student will be assessed in their ability to utter “house” in 5 trials can be framed as selecting 5 items “at random” from ${\Omega }_{1}$. “At random” is placed in quotes because the trials are not actually selected at random in the probabilistic sense, but purposefully according to some thoughtful variation of the task. The point is that these items are ideally exchangeable, so that any item selected from this space would function practically the same in testing the student’s mastery level of ${\Omega }_{1}$.

Next, we define student space $S$ as the totality of all knowledge and skills possessed by the student on all topics at a given point in time. Simply put, it is everything that the student knows about everything. Thus, the elements of $S$ are some uncountably many ordered pairs $({\Omega }_{i},{p}_{i} )$, where ${\Omega }_{i}$ is the DTT skill component space and ${p}_{i}$ is the mastery level of the student over that space. Technically, ${p}_{i}$ is the probability that student $S$ can accomplish an item selected from ${\Omega }_{i}$. It is important to differentiate ${p}_{i}$ from the performance criterion; ${p}_{i}$ represents the actual mastery level of the student, which is an intangible and exactly unknowable property. For example, for ${\Omega }_{1}$, ${p}_{1}$ represents the likelihood that the student will be able to utter the word “house” in the correct context under conditions defined by ${\Omega }_{1}.$ Thus, a student $S$ who has$({\Omega }_{1},{p}_{1}=1 )$, is someone who has completely mastered the task of uttering “house” and can do this correctly every time. In contrast, the performance criterion is some tangible, known number that is preset by the practitioner which we define as $\tau$. For example, a practitioner can set a performance criterion of $\tau =80\%$ in 10 trials. Which means that they will consider that the student has mastered the task if the student gets 8 out of 10 trials correctly. If we define the number of trials as $n$ and the number of correct responses as $x$, then the practitioner is making the inference that if $x/n\ge \tau$, then $p\ge {p}^{*}$, where ${p}^{*}$ is some desired value for actual mastery. Suppose we set ${p}^{*}=70\%$, then $\tau =80\%$ would mean that the practitioner believes that if the student is able to correctly utter “house” 80% of the time during assessment, then even if they keep providing the student with more trials, the student will be able to respond correctly to those as well at least 70% of the time. That is, an observed mastery of $\tau$ implies an actual mastery of at least ${p}^{*}$.

Therefore, the core of MIEBL’s purpose is enabling the practitioner to select a proper $\tau$ to have confidence that a student reaching this criterion has a true mastery $p$ that is at least as good as some desired ${p}^{*}$. In the language of probability, this seeks to answer the question of $\text{Pr}(p\ge {p}^{*}| x/n\ge \tau )=b$; what is the probability that the true mastery is at least the desired mastery level given that the data shows the student meeting the performance criterion? This is what is known as a Bayesian inference question (Held & Sabanés Bové, 2020). A technical discussion on how to compute these probabilities is provided in the supplementary materials.

In Table 1, these quantities are computed for a desired mastery level ${p}^{*}=90\%$ using different numbers of trials $n$. This table demonstrates how each performance criterion choice reflects confidence regarding the inference that the student’s true mastery level is 90%. The results demonstrate that when $n=5$, a performance criterion of 80% is not meaningful, as even if the student reaches this, the Bayesian inference states that the probability their actual mastery is at least 90% is only 0.20. Pragmatically, if a practitioner is aiming at such a high level of actual mastery at $n=5$, a criterion of 100% makes more sense. This allows the practitioner to infer that if a student reaches this criterion, the Bayesian probability that their actual mastery $p\geq90\%$ is 0.71.

**Table 1:** Bayesian probabilities indicating mastery of a skill component space for p* = 90%

	n = 5		n = 8		n = 10	
$x$	$\tau$	$b$	$\tau$	$b$	$\tau$	$b$
0		0.00		0.00		0.00
1		0.00		0.00		0.00
2		0.00		0.00		0.00
3		0.03		0.00		0.00
4	80%	0.20		0.00		0.00
5	100%	0.71		0.01		0.00
6			75%	0.09		0.00
7			88%	0.35		0.03
8			100%	0.81	80%	0.14
9					90%	0.44
10					100%	0.86

As such, MIEBL is very dynamic in terms of the choices that practitioners make. It does not seek to dictate what criterion should be used but provides a quantified description of the consequences of selecting each criterion in practice. Table 1 shows that for attempting to determine if a student reaches a true mastery level of 90%, a larger number of trials is needed. Suppose that the practitioner is willing to accept a true mastery standard of 70%, then the probabilities are recomputed with results shown in Table 2. Lowering the desired mastery level from 90% to 70% predictably makes every performance criterion better at supporting the decision that this new true mastery level is achieved. Thus, a practitioner who is willing to probe for a true desired mastery level of at least 70% instead of 90% can choose a performance criterion of 90% for $n=10$ and be confident that the Bayesian probability that a student meets a true mastery level of at least 70% given than they get at least 9 out of 10 trials correctly is 0.93. If this is not sufficient confidence, then the practitioner can opt for a performance criterion of 100% and have a Bayesian probability of 0.99.

**Table 2:** Bayesian probabilities indicating mastery of a skill component space for p* = 70%

	n = 5		n = 8		n = 10	
$x$	$\tau$	$b$	$\tau$	$b$	$\tau$	$b$
0		0.00		0.00		0.00
1		0.01		0.00		0.00
2		0.08		0.00		0.00
3		0.30		0.03		0.00
4	80%	0.66		0.11		0.02
5	100%	0.95		0.31		0.09
6			75%	0.60		0.24
7			88%	0.86		0.48
8			100%	0.98	80%	0.75
9					90%	0.93
10					100%	0.99

## MIEBL Tutorial

The following tutorial provides practitioners with step-by-step guidance on how they might apply MIEBL in their practice. This includes software that can be freely accessed from the included supplementary materials. The package consists of three functions: *miebl*, *miebl_re*, and *miebl_cp*. Their usage are described with examples as follows.

*miebl* is the main function that provides details like those in Table 1 and Table 2 but for practitioner-specified values of $n$ and $p$. For example, *miebl*(n = 10, tr = 0.90) provides output identical to the last column in Table 1.

*miebl_re* is a reporting function that uses output from *miebl* to provide more detailed information on a specific performance criterion of interest. For example, for *miebl*(n = 10, tr = 0.90), suppose we are interested in using a performance criterion of 80%, then we can store the result of *miebl*(n = 10, tr = 0.90) and feed it to *miebl_re* as follows:

- output1 = miebl(n = 10, tr = 0.90)
- report1 = miebl_re(mb = output1, mc = 80)

**Fig. 1:**
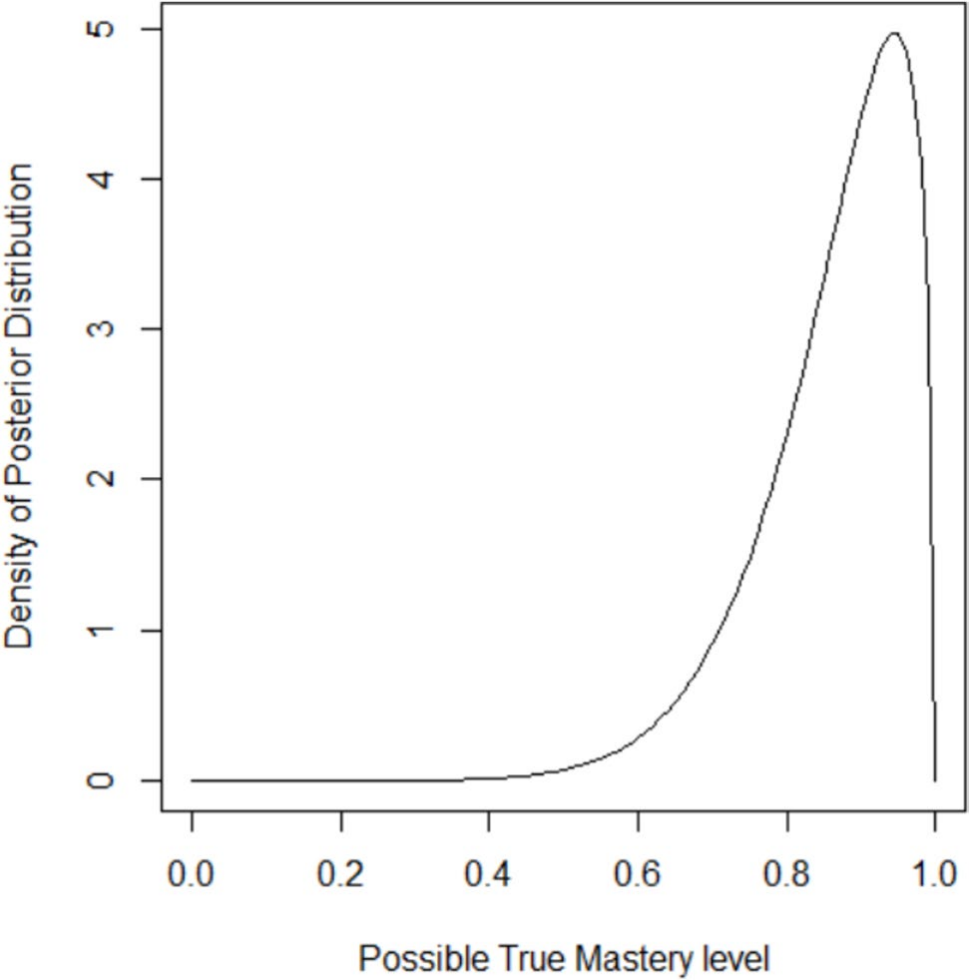
Distribution of true mastery level given the student reached a performance criterion of 90% in *n* = 10 trials

This provides the following report as well as a plot of the distribution of true mastery level given the data similar to Fig. 1.

[1]"Performance criterion of 80% (8 out of 10 items)".

[2]"If the student meets this criterion, then:"

[3]"The probability that the true mastery is at least 90% is 0.143."

[4]"There is a 95% chance that the true mastery is at least 54.75%."

[5]"The average mastery of comparable students reaching this criterion is 77.27%."

This report provides the practitioner with detailed guidance on the consequences of selecting an 80% performance criterion for the activity set. The true mastery parameter in [3] is customized by the tr argument in *miebl*, while the chance parameter in [4] is customized by the a argument in *miebl* (default is a = 0.05 for 95% chance). Thus, suppose we want to see the report for the same criterion but considering 80% true mastery (instead of 90%) and 90% chance in [4] instead of 95% then:

- output2 = miebl(n = 10, **tr = 0.80**, **a = 0.10**)
- report2 = miebl_re(mb = output2, mc = 80)

[1]"Performance criterion of 80% (8 out of 10 items)".

[2]"If the student meets this criterion, then:"

[3]"The probability that the true mastery is at least 80% is 0.4665."

[4]"There is a 90% chance that the true mastery is at least 60.52%."

[5]"The average mastery of comparable students reaching this criterion is 77.27%."

Note that statement [5] does not change since we are referring to the same posterior distribution of true mastery due to using the same performance criterion and number of trials.

Finally, *miebl_cp* allows practitioners to compare different performance criteria and different numbers of trials. The function accepts inputs from *miebl_re*, requiring a minimum of 2 and allowing up to a maximum of 5. For example, suppose the practitioner wants to compare the true mastery distributions for $n=10$. When using 80%, 90%, and 100% performance criteria, then the code is as follows:

- output = miebl(n = 10) #no need to enter tr, default is 0.90 but it is not needed
- report1 = miebl_re(mb = output, mc = 100)
- report2 = miebl_re(mb = output, mc = 90)
- report3 = miebl_re(mb = output, mc = 80)
- miebl_cp(report1, report2, report3)

**Fig. 2:**
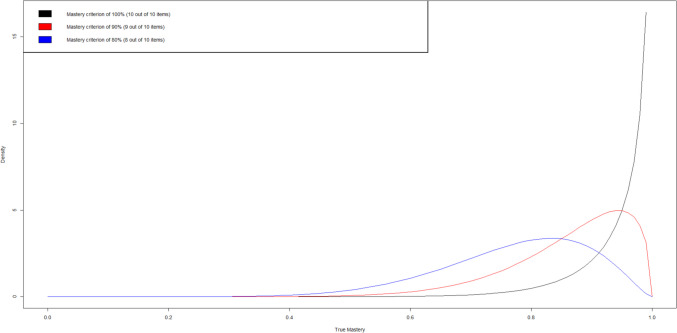
Posterior distributions of true mastery given student reached 100%, 90%, or 80% performance criteria for *n* = 10 trials

This creates the plot in Fig. 2 that compares the posterior distribution of true mastery for students reaching each of the performance criteria considered. This visual can provide further comparative guidance on which criterion to select for the task. Ultimately, the decision is still left to the practitioner; these tools only seek to provide as much information as possible to help in making that decision.

## Discussion

MIEBL provides an explicit structure for selecting performance criterion that draws justification from probabilistic statements made about the unobservable, true mastery level of a student. Current research studies in DTT use observed performance criterion as the basis for expecting that the student would be able to perform at similar levels during maintenance, and account for any decrease observed during maintenance as a measure of skill loss (Pitts & Hoerger, 2021; Wong et al., 2022a, 2022b). This implicitly equates observing a performance criterion of $\tau$ to mean that the student’s true mastery level $p$ at that point is also $\tau$. Results from MIEBL demonstrate that this is typically not the case. For example, even if a student meets a performance criterion of 90% on 10 trials (9 out of 10), the Bayesian probability that their actual mastery at that time is at least 90% is only 0.44, less than a coin toss! The distribution of possible true mastery levels is given by Fig. 1. From this distribution, the Bayesian point-estimate of the student’s mastery level in this scenario is 86%. Thus, in conducting research, assuming students who achieve a performance criterion of 90% at the end of instruction also have a true mastery of 90% and then attributing difference observed during maintenance to a durability impact is problematic. More realistically, the true mastery level at the end of assessment was somewhere less than 90% (despite observing the student reach the 90% performance criterion), and the difference observed during maintenance is a function of this bias between $\tau$ and $p$, the variance of outcomes during maintenance, and the actual loss. Recognizing and accounting for this can improve reproducibility and generalizability of research in this area. The current handbook on DTT suggests that performance criterion be selected depending on the skill of interest, with something like recognizing colors not needing 100% criterion, whereas learning to look both ways before crossing a street certainly needing 100% criterion (Richling et al., 2023). With MIEBL, the practitioner is made aware that they should select a large enough number of trials alongside a 100% performance criterion to reach the actual mastery they are targeting for such crucial skills like proper street crossing. MIEBL can also be used to help inform decisions to switch instructional strategies. Ferraioli et al. (2005) recommended using 10-trial sessions and checking if the student is at least progressing at 20% or 30% mastery before deciding between continuing with or abandoning the current strategy. MIEBL refines this guidance when considering that for $n=10$, getting 2 out of 10 indicates a Bayesian probability of only 0.22 that the true mastery level is at least 20%, whereas getting 3 out of 10 indicates a probability of 0.53 and 4 out of 10, a probability of 0.79. As such, it may be strategic to set the criterion to exactly 3 instead of 2 to 3 and allow the practitioner to save time and switch strategy more efficiently. MIEBL can also be used to directly apply baseline desired criteria drawn from other studies. For example, VanDevander et al. (2024) used a survey to estimate rates of some problem behaviors among neurotypical children. These rates can be used as desired rates in MIEBL, or used as benchmarks when choosing between different criteria.

MIEBL is also consistent with various results from literature. For example, Schneider et al. (2022) reported observing similar maintenance performance for both 1-day assessment and 3-day assessment schemes, which makes sense beyond some threshold for $n$. With a large enough sample size in a 1-day assessment strategy, there might not be much difference with a 3-day strategy for estimating the same $p$. Wong et al., (2022a, 2022b) conducted an experiment with four students and a performance criterion of 100% for $n=5$ trials, with maintenance assessments showing near-perfect retained mastery for 3 out of 4 students. This is unsurprising since 100% mastery in 5 trials implies that the probability that true mastery is at least 90% is 0.71, which means that on average, about 1 out of 4 people achieving this performance criterion would be incorrectly classified as having reached a true mastery level of at least 90%.

One important assumption on the probabilities computed in MIEBL is independence among trials. This means that in administering the assessment, the outcome of each trial must not influence the outcome of other trials. This is accounted for in existing ABA literature as inter-trial interval, where a waiting period is done between each trial to prevent it from influencing the next (Booth & Keenan, 2018). However, suppose it is of interest to account for dependence in the outcomes, more complicated distributions can be used instead of the ones used in the existing version of MIEBL to still compute the desired probabilities. This is considered as potential future work for this study. Similarly, as found from the studies reviewed on DTT, there are other ways used to infer about mastery other than computing if the proportion of successful responses meets the performance criterion. In the systematic review conducted by Wong et al., (2022a, 2022b), 83.4% of the 163 studies used the procedure considered in this study, but 4.9% used performance criterion for a consecutive number of correct trials, while 6.1% used correct response on the first opportunity. These correspond to other distributions, which can likewise be applied to MIEBL.

## Conclusion

DTT is a well-established instructional strategy, especially for helping children with autism and other developmental disabilities, but the explicit rationale for selecting performance criterion in DTT has remained vague until now. MIEBL does not advocate for a specific performance criterion but seeks to equip practitioners and researchers with a straightforward, easy-to-use tool for selecting appropriate performance criterion depending on aspects of their assessment strategy and the specific skill that they are seeking to measure. Using MIEBL, a practitioner can select the performance criterion that corresponds best to the minimum level of true mastery they want the student to have immediately after reaching the criterion. MIEBL can be expanded to encompass more assessment strategies and incorporate more distributions and distributional assumptions.

## Supplementary Information

Below is the link to the electronic supplementary material.

Supplementary file1 (DOCX 22 kb)

## Data Availability

Data sharing is not applicable. We do not analyze or generate any datasets. The work is theoretical and mathematical with proposed applications. Computer code for such applications is provided in supplementary materials.
