# Supplementary material for: MIEBL: Measurement of Individualized, Evidence-Based Learning Criteria Designed for Discrete Trial Training
Source: Behav Anal Pract. 2025 Apr 23;18(4):1283–9. doi: 10.1007/s40617-025-01058-9 (PMC12779793; doi:10.1007/s40617-025-01058-9)
Supplement: Supplementary file 1 — Supplementary file1 (DOCX 22 kb) [file 40617_2025_1058_MOESM1_ESM.docx]

Supplementary Materials

*Computation of Probabilities*

The exercise of conducting $n$ independent trials to test mastery and taking the number of correct responses $x$ out of $n$ is characterized as a binomial experiment. This allows for computation of frequentist probabilities to be straightforward using the probability mass function for a binomial distributed variable.

$$\Pr\left( X=x \right)=\binom{n}{x}p^{x}{(1-p)}^{n-x}$$

For Bayesian probability, we likewise recognize the context as a binomial experiment, but it is necessary to assume a distribution for the prior of $p$. We assume Jeffreys prior (Beta(0.5,0.5)) which assumes that the student either already knows the content, or completely does not know the content. Using this prior, we compute the posterior using Bayes Theorem. For this context, the posterior distribution is a Beta distribution with parameters $0.5+x$ and $0.5+n-x$. This is also commonly referred to as the Beta-Binomial posterior. Bayesian probabilities are computed from the posterior as follows.

$$\Pr\left( P\leq p \right)=\int_{0}^{p} \frac{y^{x-0.5}{(1-y)}^{n-x-0.5}}{B(0.5+x,0.5+n-x)}dy$$

Where B is the Beta function.

*Making Inference*

From a Bayesian perspective, to make an inference that the true mastery $p$ is at least $p^{*}$, we compute the probability that $p\geq p^{*}$ under the posterior distribution.

*Software Package for MIEBL*

*Instructions*

*Step 1: Download R and R studio*

[*https://www.r-project.org/*](https://www.r-project.org/)

[*https://posit.co/download/rstudio-desktop/*](https://posit.co/download/rstudio-desktop/)

*Step 2: Open R Studio*

*Step 3: Copy and paste the code below to R-Studio*

*Step 4: Refer back to our tutorial section*

*#Code:*

*miebl<-function(n,tr=0.90,shape1=0.5, shape2=shape1, a=0.05){*

*out<-numeric(n+1)*

*for(i in 0:n){*

*out[i+1]<-1-pbeta(tr,shape1+i,shape2+n-i)*

*}*

*freqx<-1-pbinom(c(0:n),n,tr)+dbinom(c(0:n),n,tr)*

*freqx3<-pbinom(c(0:n),n,tr)*

*freqx4<-qbeta(a,c(0:n),n-c(0:n)+1)*

*mc<-round(c(0:n)/n*100,2)*

*shape1_x<-shape1+c(0:n)*

*shape2_x<-shape2+n-c(0:n)*

*out<-as.data.frame(cbind(c(0:n),mc,round(out,4),shape1_x,shape2_x))*

*inputs<-data.frame(tr,shape1,shape2,a)*

*colnames(out)<-c("# Correct","Performance Criterion","b","post_s1","post_s2")*

*return(list(out,inputs))*

*}*

*miebl_re<-function(mb,X=nrow(mb)-1,mc=100){*

*rt<-min(X,ceiling(mc/100*(nrow(mb[[1]])-1)))*

*mx<-rt/(nrow(mb[[1]])-1)*100*

*rx<-mb[[1]][mb[[1]]$`# Correct`==rt,]*

*am<-round(rx$post_s1/(rx$post_s1+rx$post_s2),4)*

*st1<-print(paste0("Performance criterion of ",mx,"% (",rt," out of ",(nrow(mb[[1]])-1)," items)"))*

*print("If the student meets this criterion, then:")*

*st2<-print(paste0("The probability that the true mastery is at least ",mb[[2]]$tr*100,"% is ",rx$b,"."))*

*st4<-print(paste0("There is a ",(1-mb[[2]]$a)*100,"% chance that the true mastery is at least ",round(qbeta(mb[[2]]$a,rx$post_s1,rx$post_s2)*100,2),"%."))*

*st5<-print(paste0("The average mastery of comparable students reaching this criterion is ",am*100,"%."))*

*curve(dbeta(x,rx$post_s1,rx$post_s2),ylab="Density",xlab="True Mastery",main="Distribution of True Mastery given Performance Criterion is reached")*

*freport<-rbind(st1,st2,st4,st5)*

*ppost<-function(y){return(1-pbeta(y,rx$post_s1,rx$post_s2))}*

*dpost<-function(y){return(dbeta(y,rx$post_s1,rx$post_s2))}*

*return(list(freport,ppost,dpost))*

*}*

*miebl_cp<-function(R1,R2,R3=NULL,R4=NULL, R5=NULL){*

*print("Begin with the highest criterion first")*

*curve(R1[[3]](x),ylab="Density",xlab="True Mastery")*

*curve(R2[[3]](x),add=TRUE,col="red")*

*lg<-c(R1[[1]][1],R2[[1]][1])*

*cl<-c("black","red")*

*if(!is.null(R3)){*

*curve(R3[[3]](x),add=TRUE,col="blue")*

*lg<-c(R1[[1]][1],R2[[1]][1],R3[[1]][1])*

*cl<-c("black","red","blue")*

*}*

*if(!is.null(R4)){*

*curve(R4[[3]](x),add=TRUE,col="green")*

*lg<-c(R1[[1]][1],R2[[1]][1],R3[[1]][1],R4[[1]][1])*

*cl<-c("black","red","blue","green")*

*}*

*if(!is.null(R4)){*

*curve(R5[[3]](x),add=TRUE,col="pink")*

*lg<-c(R1[[1]][1],R2[[1]][1],R3[[1]][1],R4[[1]][1],R5[[1]][1])*

*cl<-c("black","red","blue","green","pink")*

*}*

*legend(x = "topleft", box.lwd = 2 ,*

*legend=lg,*

*fill = cl)*

*}*
